# Emotion dysregulation in anorexia nervosa and borderline personality disorder: Overlaps and distinctions

**DOI:** 10.1192/j.eurpsy.2026.10378

**Published:** 2026-07-31

**Authors:** K. Kucharska

**Affiliations:** The Institute of Psychology, Cardinal Stefan Wyszynski University, Warsaw, Poland

## Abstract

**Introduction:**

Altered emotion regulation (ER) plays a crucial role in the development and maintenance of psychopathology across various mental disorders, including anorexia nervosa (AN) and borderline personality disorder (BPD) (Aldao *et al*.Clinical Psychology Review, 2010; *30* 217–237). While some authors propose a transdiagnostic role for ER in mental disorders (Cludius *et al.* Emotion 2020; 20 37–42), others emphasize the importance of considering both its transdiagnostic and disorder-specific features (Aldao *et al.*Process. Cognitive Therapy and Research, 2016;40 257–261).

**Objectives:**

The aim of examining the effectiveness of cognitive reappraisal in clinical groups was expanded to include the evaluation of a maladaptive strategy—emotion suppression. Additionally, the fMRI study applied a novel set of emotion-provoking images tailored to the specific sensitivities of patients with AN and BPD.

**Methods:**

Women with BPD (N = 44), AN restrictive type (N = 38), and healthy control women (HCs; N = 40) completed clinical scales (HADS; EAT-26; BDP Checklist, and SCID-5-SPQ) and self-report measures (ED-Scale, and ERQ). fMRI tasks were conducted involving cognitive reappraisal and emotion suppression using disorder-specific stimuli. Based on the collected data, a region of interest (ROI) analysis was conducted, focusing on fronto-limbic structures involved in emotion regulation. A whole-brain analysis was also conducted, both with and without the between-group variable, to illustrate overall task-related brain activity.

**Results:**

Different neural patterns of emotion regulation (ER) were found in individuals with BPD and AN. In response to disorder-specific stimuli, BPD patients showed increased activation in the anterior orbitofrontal cortex (aOFC) and the dorsal anterior cingulate cortex (dACC) compared to the control group. No differences were found between AN patients and other groups in the ROI analysis. However, whole-brain analysis in the AN group revealed increased activity in the right angular gyrus and the cingulate cortex/precuneus, along with decreased activation in the left middle insula and the inferior frontal gyrus during cognitive reappraisal compared to controls. Across all groups, emotional state worsened following the fMRI task, with BPD and AN group reporting stronger negative affect than controls, possibly reflecting greater emotional burden.

**Conclusions:**

The discovery of distinct neural patterns of emotion regulation in AN and BPD patients highlights the complexity of emotional dysregulation mechanisms in these conditions.

**Funding:**

National Science Centre, Grant/Award Number: 2019/33/ B/HS6/03009.

**Disclosure of Interest:**

None Declared

